# Quantifying bioenergy potential and environmental impacts of agricultural residues; a Comparative analysis with global benchmarks

**DOI:** 10.1371/journal.pone.0333492

**Published:** 2025-09-30

**Authors:** Amir Azizpanah, Morteza Taki

**Affiliations:** 1 Department of mechanics Biosystem, College of Agriculture, Ilam University, Ilam, Iran; 2 Department of Agricultural Machinery and Mechanization Engineering, Faculty of Agricultural Engineering and Rural Development, Agricultural Sciences and Natural Resources University of Khuzestan, Mollasani, Iran; Nuclear Science and Technology Research Institute, IRAN, ISLAMIC REPUBLIC OF

## Abstract

Finding sustainable solutions for biofuel production from agricultural waste is crucial for reducing pollution and enhancing waste management. Agricultural residues—including crop waste, livestock manure and other organic materials—can be effectively converted into biofuels. Given the substantial volume of agricultural residues and their inadequate management in Iran, evaluating the potential for bioenergy production and determining the distribution of this renewable resource, is essential. This study assessed the bioenergy production potential from agricultural residues in Khuzestan Province, Iran. Data were collected from library resources and crop statistics for the agricultural year 2023–2024. The results indicated that the annual production of field and horticultural crops totaled 13,907,333.42 tons. The Theoretical Biomass Potential (TBP), Theoretical Energy Potential (TEP) and Available Energy Potential (AEP) were calculated as 5,496,297 tons, 92,283,088 GJ and 14,582,753 GJ, respectively. The potential electrical energy and power output from the total residues of the studied crops in a Combined Heat and Power (CHP) system were estimated at 1,364,122 MWh and 194.8745769 MWh, respectively. Additionally, the thermal energy potential was found to be 1,562,555.5 MWh, with a power capacity of 223.22 MWh. Environmental analysis indicated that the Global Warming Potential (GWP) was estimated at 774,334.45 tons of CO₂-equivalent.

## 1. Introduction

Biofuel production from agricultural waste represents a sustainable, environmentally friendly solution for reducing reliance on fossil fuels and improving waste management [1,2]. Agricultural residues—including hay, sugarcane bagasse, fruit peels and wood residues—can be converted into bioethanol, biodiesel and biogas through various processes such as hydrolysis, fermentation, pyrolysis and gasification [3–5]. This approach offers multiple advantages, including pollution reduction, renewable resource recycling, and the creation of added value from agricultural waste [3,6]. Renewable energies are compatible with natural systems, produce minimal environmental pollution and help prevent the mass emission of Greenhouse Gases (GHGs), which impose substantial costs on society [1,7]. Agricultural biomass can be converted through various energy generation technologies into different forms of energy, including electricity, heat and fuel [8]. Pollution reduction and productivity enhancement are the primary objectives of sustainable energy production [9,10]. Biomass utilization reduces emissions of sulfur dioxide (SO₂), nitrogen oxides (NO_X_) and other air pollutants compared to fossil fuels [11,12]. Energy demand and associated environmental challenges represent growing concerns in the contemporary world [13]. Although biomass accounts for 50% of renewable energy resources, it contributes only 1% to the global energy supply [3,14].

A fundamental application of crop residues, including straw and waste from crops such as wheat and barley, involves their conversion into bioenergy, particularly bioethanol and biogas [15,16]. Bioethanol serves as an important alternative to fossil fuels, representing a novel, environmentally friendly fuel that has garnered significant research interest [17,18]. Biogas has long attracted attention due to its accessibility and minimal technological requirements [19,20]. Its advantages include convertibility into various energy forms such as electricity and liquid or gaseous fuels, as well as its broad consumer base ranging from small households—particularly in rural areas—to small, medium and large-scale industrial and commercial units [21,22].

To stabilize the global climate and prevent further warming, GHG emissions—particularly CO₂—must be reduced by over 70% by 2050 [23,24]. Biomass is projected to supply nearly 38% of global fuel consumption and 17% of global power generation by 2050 [25–27]. Countries lacking petroleum resources tend to prioritize biomass development, while oil-rich nations such as the US and Canada have also made substantial investments in this field [28]. Approximately 44.1% of total global biofuel production originates from North American countries [29,30]. Given its strategic importance, the US is developing 160 plants for biofuel generation from agricultural waste [31,32]. It is estimated that farmers in Central Asian and developing countries annually burn approximately 40% of their agricultural residues [33–35].

Iran ranks among the top ten countries globally in terms of oil and gas reserves, with over 98% of its energy supplied from oil, gas, and fossil fuel sources [36–38]. Given the high potential and diversity of biomass resources in Iran, energy extraction from biomass has traditionally been prevalent. The energy potential of biomass in Iran—comprising agricultural waste (59%), animal waste (28%), urban degradable solid waste (11%), and urban and industrial degradable wastewater (2%)—was estimated in 2004/5 at approximately 132.5 million tons of crude oil equivalent [39]. Various studies have quantified the potential for bioenergy generation from agricultural waste in Iran and globally, as reviewed below. Researchers have identified agricultural waste suitable for biofuel production in Iran, reporting that the country possesses significant biofuel generation potential given its 0.86% share in global grain production and 0.9% share in global agricultural waste production. Of the 54.34 million tons of waste produced from 11 major crops in Iran, approximately 11.2 million tons can be collected for biofuel production, enabling the generation of 3.15 billion m^3^ of biogas [40]. In the US, approximately 42% of grain corn production is utilized to produce 49 billion liters of bioethanol, reducing gasoline demand by approximately 10%. According to national US projections, biofuel-based vehicle fuel production was expected to increase from 34 billion liters annually in 2007–106 billion liters in 2022 [41,42]. FAO data indicates that China, the US, Brazil, Indonesia, France, Canada, Germany, Argentina, Thailand, Iran, and the Netherlands represent leading global producers of grains and agricultural waste [43,44]. Research in the Netherlands reported total biofuel production equivalent to 1,445 million tons of crude oil in 2014 [45,46].

A five-year study assessing bioenergy production potential from agricultural waste in Markazi Province, Iran, found total annual agricultural and horticultural crop production of 1,194,134 tons, with theoretical biomass production of 910,069 tons, theoretical energy potential of 15,424,483 GJ, and extractable energy from waste of 3,340,869 GJ. Additionally, the global warming potential index was estimated at 181,954.54 tons of CO₂ equivalent [47]. Research in Southern Italy estimated annual crop residues at approximately 820,000 tons, suitable for combined power and heat generation [48]. A study on energy generation potential from agricultural waste in Bolivia reported that agricultural residues from major crops—sugarcane, soybeans, corn, sorghum, and sunflower—totaled 38 million tons, capable of generating 3.2 million tons of energy, including 4.5 TWh with a capacity of 722 MW [49]. **Table 1** assessed some related studies.

**Table 1:** Comparative overview of global studies on bioenergy potential from agricultural residues.

Location	Annual Residue (tons)	TBP (tons)	TEP (GJ)	AEP (GJ)	GWP (tons CO₂-eq)	Key Crops	Reference
Markazi, Iran	1,194,134	910,069	15,424,483	3,340,869	181,955	Wheat, barley, sugar beet	[47]
Turkey	54,036,000	–	908,119,000*	298,955,000*	–	Hazelnut, olive, grape	[50]
Argentina	204,536	–	2,605,000*	–	–	Soybean, corn, sunflower	[51]
Southern Italy	820,000	–	–	–	–	Olive, citrus, grape	[48]
Bolivia	38,000,000	–	–	–	–	Sugarcane, soybean, corn	[49]
Netherlands	–	–	1,445,000,000†	–	–	Grain, fodder crops	[45]
India (Punja)	62,500,000	–	1,050,000,000	–	1,200,000	Rice, wheat, sugarcane	[52]
China (Henan)	88,000,000	–	1,320,000,000	–	950,000	Corn, rice, wheat	[53]

*TEP/AEP in TJ (converted to GJ: 1 TJ = 1,000 GJ); †Biofuel production in crude oil equivalents; **TBP**: Theoretical Biomass Potential; **TEP**: Theoretical Energy Potential; **AEP**: Available Energy Potential; **GWP**: Global Warming Potential.

Iran, and particularly Khuzestan Province, exhibits significant potential for bioenergy production due to its rich agricultural output and favorable agro-climatic conditions. The province’s extensive cultivation of both field and horticultural crops results in substantial quantities of biomass residues, which represent a promising renewable energy resource. Given the growing need to transition away from fossil fuels, harnessing energy from agricultural waste offers a sustainable and environmentally sound alternative. Assessing the quantity and spatial distribution of this biomass is crucial for effective planning and utilization of bioenergy systems. Moreover, converting agricultural residues into energy can play a key role in GHG emissions, minimizing waste management challenges, and supporting national energy security. As the leading agricultural region in Iran, Khuzestan is strategically positioned to serve as a model for large-scale, residue-to-energy initiatives, contributing meaningfully to the country’s sustainable development and climate change mitigation goals.

## 2. Materials and methods

### 2.1. Geographical location of the studied region

Khuzestan Province is the largest province in western Iran, encompassing an area of 64,236 km^2^—representing 4% of the country’s total area—and is situated between longitudes 47°41′ to 50°39′ E and latitudes 29°58′ to 33°04′ N. The province comprises 23 counties, 51 urban districts, 53 cities, 130 rural districts, and 6,315 villages.

### 2.2. Data on agricultural and horticultural crops in the studied region

Data for this research were collected from the Agricultural Jihad Organization of Khuzestan Province, the Renewable Energy and Energy Efficiency Organization, library documents, and statistical crop annals for 2022. The average annual cultivation area for field crops in this province is approximately 115,000 ha, producing about 17 million tons of crops. The average annual cultivation area for horticultural crops is approximately 344,000 ha, yielding nearly 473,000 tons. The main horticultural crops examined in this study included date, orange, lemon, sour orange, grape, almond, walnut, pomegranate, apple, peach, apricot, olive, and fig. The primary field crops investigated comprised irrigated wheat, rainfed wheat, irrigated barley, rainfed barley, irrigated pea, rainfed pea, bean, sugar beet, canola, potato, and tomato

### 2.3. Theoretical biomass potential (TBP)

Biomass is a renewable resource composed of biological materials including forests and forest waste, crops and crop residues, horticultural and food industry by-products, animal waste, urban and industrial wastewater, sewage, and municipal solid waste. Theoretical biomass refers to the total biomass annually produced from crop residues, which varies based on crop yield, Residue-to-Product Ratio (RPR), and product moisture content (M). It is calculated using [54–55]:


$$TBP=\sum_{I=\text{1}}^{n}CP(i)\times RPR(i)\times \left[\frac{\text{1}\text{0}\text{0}-M(i)}{\text{1}\text{0}\text{0}}\right]$$ (1)

in which *TBP* is the theoretical biomass potential, or the waste derived from n products with counter i (t/year), *CP* is the annual crop production or crop yield (t/ha), *M* is the crop moisture (%), and *RPR* is the residue to crop yield ratio. In this research, RPR was estimated by Eq. (2) as an index related to Harvest Index (HI) [56]:


$$RPR=\left[\frac{\text{1}-HI}{HI}\right]$$ (2)

HI is calculated as the ratio of crop yield (e.g., grains, tubers, or fruits) to biological yield (representing the weight of all plant parts). **Table 2** presents its value for the studied crops. It should be noted that horticultural residues refer to the cellulose-rich residues from fruit tree pruning.

**Table 2:** Average harvest index of the studied crops.

Crop	Harvest Index (HI)	Source
Irrigated Wheat	44	[56]
Rainfed Wheat	35	[56]
Irrigated Barley	42	[56]
Rainfed Barley	36	[56]
Irrigated Chickpea	45	[56]
Rainfed Chickpea	42	[56]
Bean	42	[56]
Sugar Beet	79	[47]
Canola	28	[57]
Potato	75	[47]
Tomato	77	[47]
Melon	66	[47]
Watermelon	65	[47]
Cotton	30	[58]
Grain Corn	50	[47]
Rice	45	[47]
Sugarcane	66	[47]

### 2.4. Theoretical energy potential (TEP)

Theoretical energy potential derived from solid waste depends on various factors, including the chemical composition of the waste, moisture, carbon content, and its heat value. It is calculated by Eq. (3) [55]:


$$TEP=\sum_{I=\text{1}}^{n}TBP(i)\times LHU(i)$$ (3)

where TEP represents the theoretical energy potential of *n* crops with counter *i* (GJ/year), and LHV represents the lower heating value (GJ/kg).

### 2.5. Available Energy Potential (AEP)

Available energy potential (AEP) for field and horticultural crops is determined by Eq. (4) [55]:


$$AEP=\sum_{I=\text{1}}^{n}TEP(i)\times A(i)$$ (4)

where TEP denotes the theoretical energy potential derived from *n* crops with counter *i* (GJ/year), and *A* represents the available residue ratio (%).

### 2.6. Energy and power estimation

The application of Combined Heat and Power (CHP) production systems is recognized as an effective approach for estimating power and energy generation from crop residues. Due to organic biomass decomposition under anaerobic conditions, this process produces highly pure methane gas, which can serve as a source of clean energy. For lignocellulosic residues, such as pruned foliage from trees, thermochemical combustion-based conversion processes represent desirable technologies for energy extraction. This method utilizes the heat generated during the process to produce energy [59]. Generally, CHP systems are more economical and are therefore used more widely than alternative systems such as steam turbines, gas turbines, and steam engines for decentralized combined heat and power production [59].

RPR represents the quantity of residue generated per unit of crop yield. It varies depending on crop species, harvesting methods, and climatic conditions [56]. Lower Heating Value (LHV) refers to the amount of heat energy released during the combustion of crop biomass, expressed in gigajoules per kilogram (GJ/kg) [60]. Moisture content (M) of crops plays a critical role in biomass production and quality, directly affecting productivity, storage stability, and processing efficiency. Excessive moisture content reduces biofuel quality, whereas optimal moisture levels enhance biomass conversion processes such as combustion and biological fermentation [61]. **Table 3** presents the RPR, moisture content, LHV, and availability coefficient of field and horticultural crop residues [55]. Eqs. (5) and (6) were employed to calculate the electrical and thermal energy generated by the CHP system:

**Table 3:** RPR, moisture content, LHV and waste recovery coefficient of field and horticultural products.

Product	RPR	Moisture content (%)	LHV (GJ/ton)	Recovery coefficient (%)
**Horticultural Products**				
Apple	0.19	40	17.8	80
Peach	0.19	40	18.2	80
Apricot	0.19	40	20	80
Grape	0.42	45	18	80
Almond	0.6	35	18.2	80
Walnut	0.44	35	19	80
Pomegranate	0.19	40	17.8	80
Olive	0.4	90	19.7	80
Lemon	0.29	80	17.6	80
Orange	0.29	80	18.5	80
Date	0.15	50	17	80
Sour Orange	0.7	80	16.5	80
Fig	0.12	15	17.5	80
**Field Products**				
Irrigated wheat	1.27	13	16.7	15
Rainfed wheat	1.86	13	16.7	15
Irrigated barley	1.38	13	18.5	15
Rainfed barley	1.78	13	18.5	15
Irrigated chickpea	1.22	5	14.7	15
Rainfed chickpea	1.38	5	14.7	15
Bean	1.38	5	14.7	15
Sugar beet	0.27	75	16.6	35
Rapeseed	2.57	45	17.1	15
Potato	0.33	20	16	35
Tomato	0.3	5	15	35
Melon	0.52	6	14.3	35
Watermelon	0.54	6	14.3	35


$${\text{E}}_{\text{el}}=\frac{\sum_{I=\text{1}}^{n}AEP(i)\times \sigma \;\times \mu \;}{\text{3}.\text{6}}$$ (5)

$${\text{E}}_{\text{th}}=\frac{\sum_{i=\text{1}}^{n}\text{AEP}(\text{i})\times \;\beta \;\times \;\mu }{\text{3}.\text{6}}$$ (6)

where E_e1_ is the generated electrical energy (MWh), E_th_ is the generated heat energy (MWh), α and β are the electrical and heat efficiencies, and µ is the total efficiency of the system.

For the combined generation of electricity and heat from energy derived from crop residues, the electrical and thermal efficiencies of the CHP system using internal combustion engines were assumed to be 40% and 45%, respectively. For horticultural residues, these values were assumed to be 17% and 68%, respectively. Additionally, the total system efficiency was taken as 85% [48]. Assuming an annual operating time of 7000 hours for the CHP systems, the electrical and thermal power outputs were estimated using Eq. (7) and Eq. (8), respectively [48].


$${\text{p}}_{\text{el}}=\frac{{\text{E}}_{\text{e}l}}{t}$$ (7)

$${\text{p}}_{\text{th}}=\frac{{\text{E}}_{\text{th}}}{t}$$ (8)

where P_e1_ is the electrical power in MW, P_th_ is the thermal power in MW, and *t* is the annual operating hours of the power plant (h).

### 2.7. Environmental pollutant emission rate and global warming potential in bioenergy production from agricultural waste

To calculate the global warming potential associated with GHG emissions from bioenergy derived from agricultural waste, the emission coefficients for CO₂, N₂O, and CH₄ were considered to be 1, 310, and 21 kg CO₂-equivalent, respectively. The total GHG emissions were thus calculated in terms of CO₂ equivalents. These coefficients are based on the gases’ capacity to absorb infrared radiation and their atmospheric lifetime. Gases with stronger thermal effects and longer atmospheric lifespans are assigned higher coefficients [62–64]. **Table 4** presents the emission coefficients for the respective greenhouse gases.

**Table 4:** Greenhouse gas emissions coefficients.

Plant type	Co_2_ (g/kWh)	N_2_O (g/kwh)	CH_4_ (g/kwh)	Reference
Natural gas	771.269	0.0036	0.016	
CHP (horticultural waste)	1797.05	0.0072	0.144	[47]
CHP (crop residue)	500	0.0005	0.019	[47]

## 3. Results

### 3.1. Theoretical Biomass Potential (TBP), Theoretical Energy Potential (TEP), and Available Energy Potential (AEP) for field and horticultural crop residues

**Fig 1:**
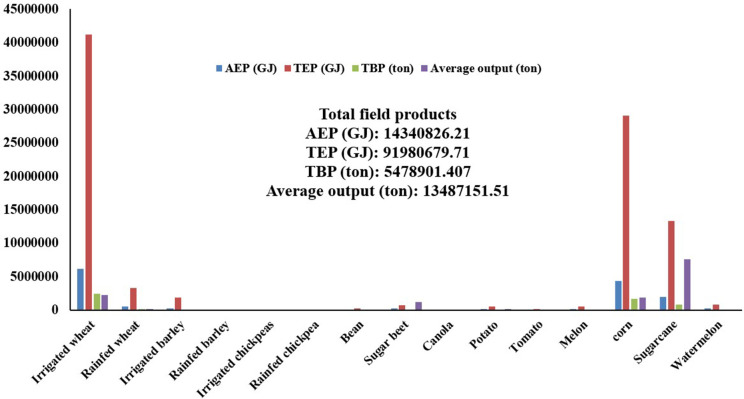
Biomass values, theoretical energy potential and energy extractable from field waste products.

**Fig 1** show the TBP, TEP and AEP of residues for average field crop productions. Biomass energy production from 15 main crops of the province was assessed using RPR and their total produced energy and AEP were estimated based on the energy value coefficient of these residues.

The average total annual crop production was estimated in this research at 13,487,151.51 tons. The main crops included sugarcane, accounting for 56.31% of total production (7,593,290.8 tons), irrigated wheat (2,233,926 tons, or 16.6%), grain corn (1,886,430.69 tons, or 13.9%), and sugar beet (1,194,089.75 tons, or 8.9%). These high production rates are associated with the fact that these crops occupy the largest cultivation areas in the province. The findings regarding biomass supply sources revealed that sugarcane, irrigated wheat, grain corn, and sugar beet have the widest cultivation areas in the province, with a total yield of 12,907,737 tons, and are thus recognized as the primary sources of biomass for sustainable energy objectives.

The main biomass sources were estimated to be irrigated wheat (2,468,264.837 tons), grain corn (1,650,626.854 tons), sugarcane (842,855.2788 tons), and irrigated barley (198,108.135 tons). The total TEP of crops in the studied region was measured at 91,980,679.71 GJ, primarily attributed to irrigated wheat, grain corn, and sugarcane, with TEPs of 41,220,022.78, 29,051,032.63, and 13,317,113.41 GJ, respectively. AEP was found to be 14,340,826.21 GJ. Researchers in Turkey investigated the energy potential of agricultural biomass residues and reported that TBP was 54,036,000 tons, TEP was 908,119 TJ, and the AEP of crop residues was 298,955 TJ [55]. A research study in Argentina revealed that a total of 204,536 tons of field and horticultural crops were produced in the country, with an energy potential of 2,605 TJ per year [51].

**Fig 2:**
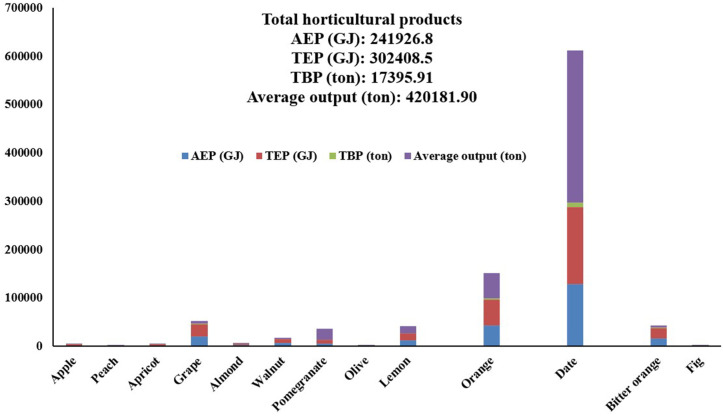
Biomass values, theoretical energy potential and energy extractable from horticultural waste products.

**Fig 2** show the TBP, TEP and AEP of residues for average horticultural productions. The total mean production of the 13 main horticultural crops—including potato, peach, apricot, grape, almond, walnut, pomegranate, olive, lemon, orange, date, sour orange, and fig—was 420,181.91 tons. TBP was estimated at 17,395.91 tons, TEP at 302,408.5 GJ, and AEP at 241,926.82 GJ. The highest production levels among these crops were associated with date (314,416.86 tons), orange (51,309.45 tons), and pomegranate (22,038.06 tons), accounting for 74.82%, 12.21%, and 5.24% of total production, respectively. A research study in Iran showed that crops such as wheat, barley, rice, corn, pistachio, sugarcane, sugar beet, canola, soybean, and date had high TBP, AEP, and TEP values. Straw and grain residues represented the largest share of biomass resources [56].

The TBP of field and horticultural crops in Khuzestan Province was found to be 5,496,297.32 tons. The most significant residues were associated with irrigated wheat, grain corn, and sugarcane among field crops, and date, orange, and grape among horticultural crops. The total TEP of horticultural and field crop residues was calculated at 92,283,088 GJ, with the contribution of field crop residues being greater than that of horticultural residues. The total AEP of horticultural and field crops was found to be 14,582,753.03 GJ, with crop residues playing a particularly noteworthy role. In a similar study conducted in Turkey, the biomass production potential from field and horticultural crops was investigated. The production potential of horticultural crops was calculated to be 15,652,000 tons, and the theoretical potential for total biomass from field and horticultural crops was estimated at 4,986,000 tons. The three leading horticultural crops in Turkey in terms of residue production (dry biomass from pruning) were hazelnuts, olives, and grapes, respectively [55]. Based on the findings from biomass energy production assessments in agriculture and the substantial cultivation area of field and horticultural crops in Khuzestan Province, the use of crop residues for energy generation can be considered a promising approach to reducing reliance on fossil fuels.

### 3.2. Sensitivity analysis

To assess the robustness of the bioenergy potential estimates for agricultural residues in Khuzestan Province, a sensitivity analysis was conducted on the RPR. The variation of ±10% and ±20% was used to evaluate their impact on TBP, TEP and AEP. The analysis focused on three major crops—sugarcane, irrigated wheat, and grain corn—due to their significant contribution to the province’s biomass production. The results, presented in **Table 5**, illustrate the effects of coefficient variations on TBP, TEP, and AEP. For sugarcane (baseline: TBP = 842,855 tons, TEP = 13,317,113 GJ, AEP = 4,661,990 GJ), a ± 20% variation in RPR results in TBP ranging from 674,284–1,011,426 tons, TEP from 10,653,690–15,981,736 GJ, and AEP from 3,730,802–5,593,608 GJ. Similar proportional changes are observed for irrigated wheat (baseline: TBP = 2,468,265 tons, TEP = 41,220,023 GJ, AEP = 6,183,003 GJ) and grain corn (baseline: TBP = 1,650,627 tons, TEP = 29,051,033 GJ, AEP = 4,357,655 GJ). These findings confirm that the baseline estimates are robust, as they remain within a reasonable range under coefficient variations. However, the linear relationship between coefficient changes and output values highlights the importance of precise coefficient data for accurate bioenergy planning. Potential uncertainties arising from crop-specific factors, regional conditions, or harvesting practices are further discussed in the discussion section.

**Table 5:** Sensitivity analysis of TBP, TEP, and AEP for key crops with ±10% and ±20% variation.

Crop	TBP (tons)	TEP (GJ)	AEP (GJ)
**Sugarcane (Baseline: TBP = 842,855 tons; TEP = 13,317,113 GJ; AEP = 4,661,990 GJ)**			
RPR (−20%)	674284	10653690	3730802
RPR (−10%)	758570	11985302	4194856
RPR (Baseline)	842855	13317113	4661990
RPR (+10%)	927141	14648725	5127074
RPR (+20%)	1011426	15981736	5593608
**Irrigated Wheat (Baseline: TBP = 2,468,265 tons; TEP = 41,220,023 GJ; AEP = 6,183,003 GJ)**			
RPR (−20%)	1974612	32976018	4946403
RPR (−10%)	2221438	37098020	5564703
RPR (Baseline)	2468265	41220023	6183003
RPR (+10%)	2715091	45362025	6804304
RPR (+20%)	2961918	49494027	7424104
**Corn (Baseline: TBP = 1,650,627 tons; TEP = 29,051,033 GJ; AEP = 4,357,655 GJ)**			
RPR (−20%)	1320502	23240826	3486124
RPR (−10%)	1485564	26145930	3921889
RPR (Baseline)	1650627	29051033	4357655
RPR (+10%)	1815689	31956136	4793420
RPR (+20%)	1980752	34861239	5229186

### 3.3. Calculation of combined electrical and thermal energy and power of the CHP system from field and horticultural crop residues

**Table 6** presents the values of electrical and thermal energy, as well as electrical and thermal power, of the CHP system based on field and horticultural crop residues studied in Khuzestan Province.

**Table 6:** Energy values and combined electrical and thermal power in the CHP system from field and horticultural waste.

Field Products	Electrical energy (MWh)	Thermal energy (MWh)	Electrical power (MW)	Thermal power (MW)
Irrigated wheat	583950.3228	656944.1131	83.42147468	93.84915902
Rainfed wheat	46869.08294	52727.71831	6.695583277	7.532531187
Irrigated barley	26730.01882	30071.27117	3.818574117	4.295895882
Rainfed barley	1617.271118	1819.430008	0.231038731	0.259918573
Irrigated chickpeas	20.65332495	23.23499057	0.002950475	0.003319284
Rainfed chickpea	9.33713865	10.50428098	0.001333877	0.001500612
Bean	4067.648008	4576.10401	0.581092573	0.653729144
Sugar beet	24452.47039	27509.02919	3.493210056	3.929861313
Canola	1066.091596	1199.353045	0.152298799	0.171336149
Potato	16976.64789	19098.72888	2.425235413	2.72838984
Tomato	5043.301813	5673.714539	0.720471688	0.810530648
Melon	17457.18967	19639.33838	2.493884238	2.805619768
Corn	411556.2955	463000.8325	58.79375651	66.14297607
Sugarcane	188659.1066	212241.4949	26.95130094	30.32021356
Watermelon	25935.92676	29177.91761	3.705132394	4.168273944
Total	1354411.364	1523712.785	193.4873378	217.673255
**Horticultural products**				
Apple	86.56481771	346.2592708	0.043282409	0.115419757
Peach	18.72138147	74.88552587	0.009360691	0.024961842
Apricot	80.27146473	321.0858589	0.040135732	0.10702862
Grape	812.8495729	3251.398292	0.406424786	1.083799431
Almond	100.8361518	403.3446071	0.050418076	0.134448202
Walnut	270.3645402	1081.458161	0.13518227	0.360486054
Pomegranate	239.3328636	957.3314543	0.119666432	0.319110485
Olive	36.21819364	144.8727745	0.018109097	0.048290925
Lemon	473.6630991	1894.652397	0.23683155	0.631550799
Orange	1729.654304	6918.617216	0.864827152	2.306205739
Date	5149.100127	20596.40051	2.574550063	6.865466836
Bitter orange	685.3914728	2741.565891	0.342695736	0.913855297
Fig	27.70564585	110.8225834	0.013852823	0.036940861
Total	9710.673635	38842.69454	4.855336817	12.94756485
Field and horticultural products	1364122	1562555.50	682.061019	520.8518265

The results showed that field crop residues could potentially produce 1,354,411.36 MWh of electricity with a power output of 193.49 MW, and 1,523,712.8 MWh of thermal energy with a power output of 217.67 MW in this province. Wheat, corn, sugarcane, and barley ranked first to fourth in electricity and heat production potential among crop residues, respectively. These crops can potentially produce 630,819.41, 411,556.29, 188,659.11, and 28,347.29 MWh of electricity, and 709,671.83, 463,000.83, 212,241.49, and 31,890.71 MWh of thermal energy, respectively. Horticultural crop residues were estimated to have the potential to produce 9,710.67 MWh of electricity with a power output of 4.86 MW, and 38,842.69 MWh of thermal energy with a power output of 12.95 MW in the studied province. The leading horticultural crops were found to be date, orange, grape, and sour orange, which are capable of producing 5,149.1, 1,729.65, 812.85, and 685.39 MWh of electrical energy, and 20,596.4, 6,918.62, 3,251.39, and 2,741.56 MWh of thermal energy, respectively.

Date is an important horticultural crop that plays a vital role in food security, job creation, environmental protection and sustainability, and the national economy in the region. It has significantly higher potential for generating electrical and thermal energy compared to other horticultural crops. Dates are currently widely cultivated in the provinces of Khuzestan, Kerman, Fars, Sistan and Baluchestan, Hormozgan, Isfahan, Yazd, Kohgiluyeh and Boyer Ahmad, Khorasan, Kermanshah, Ilam, Semnan, and Bushehr, making these provinces the primary date production areas in Iran. These regions, with their hot and dry climatic conditions, provide ideal environments for the growth and fruiting of various date varieties [63].

The results indicated that total biomass resources of field and horticultural crops in Khuzestan Province can produce 1,364,122.03 MWh of electrical energy with a power output of 194.87 MW and 1,562,555.5 MWh of thermal energy with a power output of 223.22 MW. Electricity generation and consumption are of great importance in this province, as it faces challenges in addressing energy imbalance and ensuring sustainable electricity supply. To cope with these challenges, it is essential to develop energy production resources and manage consumption. This province has high potential for generating electrical energy from biomass, given the extensive availability of biomass from its abundant natural and agricultural resources, including crop residues. Therefore, it can play an important role in sustainable energy supply, reducing dependence on fossil fuels, and contributing to economic development.

### 3.4. Environmental analysis of bioenergy production from field and horticultural crop residues

**Table 7** presents the GHG emissions and GWP associated with the generation of electrical energy from agriculture and horticultural residues.

**Table 7:** The results of GHG emissions and GWP for all the agriculture and horticultural residues.

Agricultural Products	Co_2_ (ton)	N_2_o (ton)	CH_4_ (ton)	GWP CO_2_.eq ton	Percentage of GWP
Irrigated wheat	291975.16	0.2919	11.0951	292298.6699	43.16
Rainfed wheat	23434.54	0.0234	0.8905	23460.50694	3.46
Irrigated barley	13365.01	0.0133	0.5078	13379.81784	1.97
Rainfed barley	808.63	0.0008	0.0307	809.5315274	0.12
Irrigated chickpeas	10.33	0.0000	0.0003	10.33810442	0.0015
Rainfed chickpea	4.67	0.0000	0.0002	4.6737421	0.0006
Bean	2033.82	0.0021	0.0773	2036.077481	0.31
Sugar beet	12226.23	0.0122	0.4646	12239.78187	1.81
Canola	533.04	0.0005	0.0202	533.6364125	0.079
Potato	8488.32	0.0085	0.3225	8497.72901	1.25
Tomato	2521.65	0.0025	0.0958	2524.444895	0.37
Melon	8728.59	0.0087	0.3316	8738.266117	1.29
Corn	205778.15	0.2057	7.8195	206006.15	30.42
Sugarcane	94329.55	0.0943	3.5845	94434.07043	13.94
Watermelon	12967.96	0.0129	0.4928	12982.33188	1.917
Total Emissions	677205.68	0.6772	25.734	677956.0261	100
GWP(Co_2_ eq ton)	677205.68	209.93	540.41	753633.7611	–
**Horticultural products**					
Apple	155.5613057	0.000623267	0.012465334	156.0162903	0.894047161
Peach	33.64325856	0.000134794	0.002695879	33.74165815	0.193355666
Apricot	144.2518357	0.000577955	0.011559091	144.6737425	0.829048995
Grape	1460.731325	0.005852517	0.117050339	1465.003662	8.395164132
Almond	181.2076066	0.00072602	0.014520406	181.7376014	1.041442443
Walnut	485.8585969	0.001946625	0.038932494	487.2796329	2.792342846
Pomegranate	430.0931225	0.001723197	0.034463932	431.351056	2.471845639
Olive	65.08590488	0.000260771	0.00521542	65.2762677	0.374063899
Lemon	851.1962723	0.003410374	0.068207486	853.6858455	4.892023805
Orange	3108.275267	0.012453511	0.24907022	3117.36633	17.86398401
Date	9253.190383	0.037073521	0.741470418	9280.254053	53.18024655
Bitter orange	1231.682746	0.004934819	0.098696372	1235.285164	7.078768447
Fig	49.78843087	0.000199481	0.003989613	49.93405174	0.286145742
Total Emissions	17450.56606	0.06991685	1.398337003	17501.60536	100.2924793
GWP (Co_2_ eq ton)		21.67422355	29.36507707	24786.24198	0.894047161
Total pollutions on agricultural and horticultural products	694656.2	0.747122532	27.13215	695457.6314	–
Global warming potential of agricultural and Horticultural products (Co_2_ eq ton)	694656.2	231.607985	541.3006	778420.003	–

The total GHG emissions from electricity generation using agricultural residues were calculated to be 677,956.026 t/year, with contributions from CO₂, N₂O, and CH₄ amounting to 677,205.7, 0.68, and 25.73 t/year, respectively. The GWP of energy production from these residues was estimated at 753,633.76 t CO₂-equivalents. The findings indicated that wheat had the highest GWP share from electricity production using agricultural residues, accounting for 315,759.18 t CO₂-equivalents, or 46.57% of the total. Following wheat were grain corn, sugarcane, and barley, contributing 30.38%, 13.92%, and 2.09% of the total GWP, respectively. For horticultural residues, date palm was found to contribute the most to GWP, accounting for 53.02% or 9,280.25 t CO₂-equivalents. Orange, sour orange, and lemon followed with shares of 17.81%, 7.06%, and 4.88%, respectively. Environmental assessments of cucumber and sunflower production reported GWPs of 8,554.97 and 2,417.42 kg CO₂-equivalents/ha, respectively. Corresponding CO₂ emissions were calculated as 4,997.75 and 1,129.34 kg CO₂-equivalents/ha, respectively [64]. **Table 8** reviewed the results of some related studies with present research. **Table 9** shows the indicates of analysis of variance to show the effects of sources on energy indices and GHG emissions.

**Table 8:** Comparison of bioenergy outputs and environmental impacts.

Location	Electrical Energy (MWh)	Thermal Energy (MWh)	CHP Power (MW)	GWP (tons CO₂-eq)	Key Residues	Reference
Khuzestan, Iran	1,364,122	1,562,555	194.87 (el) 223.22 (th)	774,334	Wheat, corn, sugarcane	Present study
Markazi, Iran	–	–	–	181,955	Wheat, sugar beet	[47]
Turkey	–	–	3,500	–	Hazelnut, olive	[50]
Bolivia	4,500,000*	–	722	–	Sugarcane, corn	[49]
Southern Italy	285,000	410,000	40.7	110,000	Olive pruning, citrus waste	[48]
India (Punjab)	2,100,000	–	300	1,200,000	Rice straw, sugarcane bagasse	[52]
China (Henan)	3,850,000	4,200,000	550	950,000	Corn stover, rice husk	[53]
Netherlans	–	–	–	500,000†	Grain residues	[41]

*4.5 TWh = 4,500,000 MWh; †Estimated from national biofuel production; **CHP**: Combined Heat and Power; **el**: electrical; **th**: thermal.

**Table 9:** Analysis of Variance (ANOVA) results for the effects of sources of variation on energy indices and greenhouse gas emissions.

Sources of variation	df	ss	ms	pv > F	R^2^	C. (V%)
CO_2_	8	3.30	0.41	0.04	0.85	26
N_2_O	8	0.44	0.05	0.0001	0.94	21
CH_4_	8	200.55	25.07	0.0001	0.97	30
GWP	8	160.80	20.10	0.0001	0.98	10
Electrical energy	7	36.43	5.20	0.04	0.93	26
Thermal power	7	37662.63	5380.37	0.0001	0.98	24.15
Thermal energy	7	511.46	73.06	0.0001	0.99	5.18
Electrical power	7	24367.78	3481.11	0.0001	0.98	23

The results of **Table 9** demonstrate that the identified sources of change exert a statistically significant influence on GHG emissions and key energy indicators. The high determination coefficients (R^2^ = 0.85–0.99) indicate that the model explains a substantial proportion of the observed variability, reflecting strong predictive accuracy. Furthermore, low Coefficients of Variation (C.V) for indicators such as thermal energy and GWP underscore the reliability and consistency of the results, suggesting limited dispersion around the mean values. In contrast, the elevated C.V. observed for methane (CH₄) emissions reflects greater data heterogeneity, likely driven by spatial and operational variability in environmental conditions—resulting in wider deviations from the mean. The analysis presented in **Table 9** highlights resource management as a critical lever for reducing GHG emissions and enhancing energy efficiency. These findings provide a robust foundation for designing process optimization strategies and advancing environmental sustainability in agricultural and industrial systems. To show the more details, average energy and GHG indicators were analysis by Duncan method (**Table 10**).

**Table 10:** The analysis of average energy and greenhouse gas indicators in various agricultural products by the Duncan test method.

Sources of variation	Irrigated wheat	Irrigated barley	Sugar beet	Canola	Corn	Sugarcane	Grape	Date	Orange
Electrical energy	583951^a^	26728^c^	24449^d^	1062^f^	188654^b^	821^g^	5144^e^	682^h^	–
Thermal power	91.267^a^	4.408^c^	3.560^c^	0.156^c^	29.320^b^	1.246^c^	6.090^c^	0.926^c^	–
Thermal energy	656946^a^	30080^c^	27512^c^	3702^d.^	212244^b^	3254^e^	20596^c^	2746^e^	–
Electrical power	85.171 ^a^	3.074^c^	3.338^c^	0.155^c^	24.506^b^	0.484^c^	2.627^c^	0.333^c^	–
CO_2_	291975^b^	13360^c^	12228^c^	529^c^	205775^b^	94327^c^	1458^c^	9257^c^	304921616^a^
N_2_O	0.3044^a^	0.0283^c^	0.0165^c^	0.00065^c^	0.2657^a^	0.0693^b^	0.00735^c^	0.03857^c^	0.00473^c^
CH_4_	9.5951^a^	0.4578^d^	0.4346^d^	0.0152^d^	7.3195^b^	3.3745^c^	0.1196^d^	0.8015^d^	0.0987^d^
GWP	292305^a^	13368^d^	12244^e^	530^g^	206011^b^	9444^c^	1470^g^	9275^f^	1243^g^

The Duncan multiple range test revealed statistically significant differences among agricultural crops with respect to energy consumption and GHG emissions. Irrigated wheat consistently ranked in Group a across all energy indicators—electrical energy, thermal power and thermal energy—demonstrating the highest values. Corn followed closely, assigned to Group b, and ranked second in both energy use and GHG emissions. In contrast, horticultural crops were predominantly classified in lower groups (c through g), indicating significantly reduced energy demands and emissions compared to Irrigated wheat and corn. High-input cereal crops such as irrigated wheat and corn exhibited the greatest levels of electrical energy use, thermal energy demand, and emissions of CO₂, N₂O, and CH₄. Among these, oranges recorded the highest CO₂ emissions, followed by irrigated wheat and corn. Irrigated wheat and corn also demonstrated the greatest potential for N₂O emissions, reflecting their heavy reliance on nitrogenous fertilizers. Similarly, these crops produced the highest CH₄ emissions, contributing to their elevated GWP. Specifically, irrigated wheat had the highest GWP at 292,305 CO₂-equivalent units, followed by corn at 206,011, underscoring their substantial contribution to climate change.

In terms of energy generation, irrigated wheat led in both electrical energy (583,951 MJ) and thermal energy (656,946 MJ), reinforcing its resource-intensive profile.

Conversely, horticultural crops exhibited markedly lower energy requirements and GHG emissions, highlighting their relatively lower environmental footprint. So, it can be concluded that cereal crops like irrigated wheat and corn contribute disproportionately to energy consumption and GHG emissions, primarily due to intensive inputs—including synthetic fertilizers, irrigation, and mechanization—whereas horticultural crops exhibit lower environmental impacts owing to reduced input intensity and more efficient land-use patterns.

**Table 11** presents a normalized comparison of three key agricultural commodities using standardized metrics to evaluate their relative efficiency as bioenergy feed stocks. Corn emerges as the most energy-dense feedstock at the field level, with the highest TEP at 659 GJ/ha, significantly surpassing irrigated wheat (407 GJ/ha) and sugarcane (130 GJ/ha). This underscores corn’s exceptional productivity and biomass yield potential under Khuzestan’s agro-climatic conditions. Despite its lower TEP, sugarcane remains a strategically vital crop due to its exceptionally high total annual production volume (56% of all field crop output), making it the dominant contributor to the province’s overall bioenergy potential.

**Table 11:** A comparison between three main agricultural productions based on some standard metrics.

Crop	TEP (GJ/ha)	Electrical Energy (MWh/ton yeild)	GWP CO_2_.eq ton (tons/ton yeild)
Sugarcane	130	0.02	0.01
Irrigated wheat	407	0.26	0.13
Corn	659	0.22	0.11

When evaluating efficiency per unit of residue, irrigated wheat demonstrates the highest electrical energy yield (0.26 MWh/ton yeild), followed closely by corn (0.22 MWh/ton yeild), while sugarcane lags considerably at only 0.02 MWh/ton yeild. This indicates that although sugarcane produces vast quantities of biomass, its residue has a lower calorific value or conversion efficiency compared to cereal residues, likely due to its higher moisture content and different chemical composition (e.g., higher lignin/cellulose ratios impacting combustion or gasification).

Critically, the environmental burden analysis reveals that both irrigated wheat and corn have substantially higher GWP per ton of residue (0.13 and 0.11 tons CO₂-eq/ton, respectively) compared to sugarcane (0.01 tons CO₂-eq/ton). This aligns directly with the findings from **Table 10** and the ANOVA results, confirming that the intensive nitrogen fertilizer inputs required for high-yield cereal production are the primary driver of elevated N₂O emissions, which dominate the GWP calculation. In contrast, sugarcane’s relatively low GWP footprint per ton stems from its lower dependence on synthetic nitrogen fertilizers, despite its large total emissions due to sheer volume.

## 4. Discussion

This study reveals that Khuzestan Province, Iran’s most agriculturally productive region, holds immense bioenergy potential from crop and horticultural residues. With a TEP of 92.3 million GJ/year and an AEP of 14.6 million GJ/year, the province could generate 1,364,122 MWh of electricity and 1,562,555 MWh of thermal energy annually through CHP systems—making it a key player in Iran’s renewable energy transition.

### 4.1. Regional and global comparisons

Khuzestan’s bioenergy potential is among the highest in the Middle East:

- It surpasses Markazi Province, Iran, by nearly sixfold in TEP (15.4 million GJ), due to larger cultivation areas and high-yield crops like sugarcane (56% of total output), wheat, and corn.
- Its TEP is comparable to Turkey’s national potential (90.8 million GJ) despite having far less agricultural land, highlighting exceptional agricultural intensity and residue productivity.
- While AEP is lower than in major producers like India’s Punjab (~50.4 million GJ) or China’s Henan, Khuzestan’s feedstock diversity and favorable residue calorific values (e.g., sugarcane: 17.8 GJ/ton) support efficient energy conversion.

Notably, Khuzestan uniquely integrates date palm pruning residues, contributing over 5,100 MWh of electricity potential—a rare advantage shared only with date-producing regions in Pakistan and the Arabian Peninsula.

### 4.2. Energy conversion efficiency

The province’s estimated CHP capacity of 194.87 MW (electrical) and 223.22 MW (thermal) exceeds that of Southern Italy (40.7 MW) and is competitive with Bolivia (722 MW), despite using less than half the biomass volume. This reflects high feedstock quality and optimized conversion technologies tailored to different residues:

- Field crop residues: Converted via internal combustion engines (40% electrical efficiency).
- Woody horticultural biomass: Used in combustion systems with 17–68% thermal efficiency.

### 4.3. Environmental impact and climate benefits

The GWP of bioenergy production in Khuzestan is estimated at 774,334 tons of CO₂-equivalent, primarily from indirect emissions (machinery, transport, processing). However, this represents a net reduction in greenhouse gas emissions:

- Replacing fossil fuels with bioenergy avoids 600,000–700,000 tons of direct CO₂ emissions for the same electricity output.
- The GWP intensity (569 kg CO₂-eq/MWh) is lower than in many global counterparts, indicating a climate-efficient system.

Despite this, the study focused only on GWP due to data limitations; future assessments should include acidification, eutrophication, and water footprint for a full environmental profile.

### 4.4. Limitations of environmental impact assessment

The study focuses solely on Global Warming Potential (GWP) to assess the climate change mitigation benefits of bioenergy from agricultural residues, aligning with its goal of evaluating bioenergy as a fossil fuel alternative. GWP was chosen for consistency with global studies, enabling direct comparisons. However, other environmental impacts—such as acidification, eutrophication, and water footprint—were not evaluated due to limited data and the study’s primary focus on energy potential rather than a full Life Cycle Assessment (LCA). These factors are important for a comprehensive environmental evaluation, and future research should include primary data collection and LCA to better understand the overall sustainability of bioenergy systems in Khuzestan.

### 4.5. Challenges and opportunities

Current residue recovery rates are low (15–35% for field crops, 80% for horticultural), constrained by:

- Manual harvesting practices
- Lack of mechanization
- Poor logistics and storage infrastructure

Yet, even a 50% increase in recovery could boost AEP to over 20 million GJ/year, rivaling top global bioenergy regions.

### 4.6. Uncertainties in bioenergy potential estimates

The bioenergy potential estimates (TBP, TEP, and AEP) in this study are based on fixed Residue-to-Product Ratios (RPR), but these values carry uncertainties due to crop-specific traits, regional conditions, and harvesting practices. Variations in crop varieties, growth stages, soil fertility, irrigation, and climate in Khuzestan can affect RPR and Lower Heating Value (LHV) of residues, leading to inaccuracies in biomass and energy estimates. Recovery rates also vary significantly depending on collection methods (manual vs. mechanized) and logistical challenges. Sensitivity analysis shows that ±20% changes in input coefficients result in proportional changes in energy potential estimates, highlighting the importance of using region-specific data. To improve accuracy, future research should focus on collecting primary data on local residue characteristics and harvesting efficiencies in Khuzestan.

### 4.7. Policy recommendations

To unlock Khuzestan’s potential, Iran should:

1. Incentivize mechanized collection through subsidies for harvesting equipment.
2. Invest in rural logistics, including decentralized storage and transport networks.
3. Develop bioenergy infrastructure, such as small-to-medium CHP and biogas plants in high-residue zones (e.g., Ahvaz, Dezful).
4. Support pilot projects and technology transfer from countries with mature bioenergy sectors (e.g., the U.S.).
5. Integrate bioenergy into national policy, setting targets (e.g., 5% of energy mix by 2035) and offering tax incentives.

### 4.8. Innovative contributions

This study makes several original contributions:

- First comprehensive bioenergy assessment of Khuzestan, filling a critical gap in Iran’s renewable planning.
- Inclusion of horticultural residues (especially date palm pruning), often ignored in global models.
- Standardized cross-regional benchmarking using consistent metrics (GJ, MWh, MW, CO₂-eq).
- Dual evaluation of energy potential and environmental impact.
- Realistic, feedstock-specific CHP modeling enhancing technical feasibility.

## 5. Conclusions

Considering the total biomass from field and horticultural crops in Khuzestan Province, it is evident that the TBP, TEP and AEP are significant in this region. During 2020–2021, the average annual production of these crops was approximately 13.91 million tons. Among field crops, sugarcane, irrigated wheat, corn and sugarcane were the primary contributors, while dates and oranges dominated among horticultural crops. These are key sources of biomass suitable for sustainable energy production. The TBP in Khuzestan Province was estimated at around 5.5 million tons. Crop residue TEP exceeded 92 million GJ, and the AEP was approximately 14.5 million GJ, with wheat, corn, and sugarcane contributing most significantly. From an environmental perspective, electricity generation from these residues would result in estimated GHG emissions of about 695,000 tons, with a global warming potential of roughly 774,000 tons of CO₂-equivalents. Given the substantial biomass volume and energy potential, Khuzestan Province holds strong promise as a source of clean and sustainable energy. Harnessing this potential can reduce dependency on fossil fuels, promote regional economic growth, and support environmental sustainability. Optimal utilization of this resource represents a strategic step toward expanding renewable energy and mitigating ecological impacts in the region.

## Supporting information

S1 Data (XLSX)
